# Does internal critical examination lead to improved quality of clinical practice? A retrospective follow-up analysis concerning the success rates of Myringoplasty

**DOI:** 10.1007/s00405-025-09669-2

**Published:** 2025-10-28

**Authors:** Michiel H. van Lier, Willemijn J.M. Van Diemen, Mark C.J. Aarts

**Affiliations:** 1 Departement of Otorhinolaryngology, Jeroen Bosch Hospital, Henri Dunantstraat 1, ‘s-Hertogenbosch, The Netherlands; 2 Faculty of Health, Medicine and Life Sciences, Maastricht University, Maastricht, The Netherlands

**Keywords:** Myringoplasty, Tympanic membrane perforation, Otologic surgical procedures, Health care evaluation programs, Quality improvement

## Abstract

**Purpose:**

The aim of this study was to assess whether recommendations from a retrospective study conducted in 2019 translated into measurable changes in clinical practice and patient outcomes.

**Methods:**

In this retrospective follow-up cohort study, patients who underwent a myringoplasty between January 2020 and December 2021 at the Jeroen Bosch Hospital in The Netherlands were analyzed. The same methodology was applied and the same patient, surgical, and follow-up data were collected as in our previous study in 2019. Recommendations in 2019 included a minimum of 15 procedures per surgeon per year, application of cartilage grafts, and reduction of silastic sheets use. The primary outcome was to assess the adherence to the 2019 recommendations. Secondary outcome was the change in the success rate of the myringoplasty, and tertiary outcome was the auditory performance after surgery.

**Results:**

172 patients were analyzed. While recommended in 2019, the required number of 15 procedures per year was not always reached. Additionally, use of temporalis fascia was reduced from 80 cases in 2019 to 4 in 2024. The use of silastic sheets was reduced from 80 cases in 2019 to 13 cases in 2024. The overall success rate of closure of the perforation was 88.4% compared to 74.9% in 2019. Postoperative audiometry showed significant air conduction gain in successful anatomical closure compared to unsuccessful closure (9.06 ± 8.48 dB vs. 4.14 ± 6.96 dB).

**Conclusion:**

Internal self-assessment of surgical outcomes is associated with changes in surgical behavior and subsequently increased closure success rates.

## Introduction

Critical examination of clinical practice was established as an integral component of patient care through the development of evidence-based medicine during the mid-nineties [1]. This process included the continuous evaluation of clinical processes and outcomes to improve the quality of provided care [1]. The evaluation of current practice allows an appropriate response to any variation in the standard of care provided for individual patients [1]. Effective evaluation could prompt adjustments in treatment guidelines, thus leading to improved quality of care.

Different tools to evaluate the outcomes of clinical care have been developed and applied in current health care. For instance, patient-reported outcome measures (PROMs) are frequently implemented and are being studied as a critical evaluation tool [2]. Similarly, integrated care pathways (ICPs) are structured evaluating care plans particularly useful in surgery [3]. Although these monitoring tools were developed, the question remains how local evaluation leads to adjustment of surgical behavior and better surgical outcomes. Are surgeons willing to adapt their surgical techniques according to the outcomes of these quality analyses? And does this adjustment in surgical behavior lead to improved outcomes?

In 2019 we conducted a study concerning determinants influencing myringoplasty success rates in daily clinical practice [4]. We identified several surgical factors affecting tympanic membrane closure success in myringoplasty. These included a higher failure rate with the use of temporalis fascia compared to cartilage or butterfly grafts, reduced success with the use of silastic sheets, significant outcome variability among surgeons depending on their frequency of myringoplasties performed annually, and a positive correlation between successful closure and hearing improvement [4]. According to these outcomes, practice guidelines were changed and the application of these revisions in clinical care was stimulated. The most important alterations in these guidelines included a minimum of 15 procedures per year per surgeon, the use of cartilage (underlay) or butterfly graft instead of other materials, and the abandonment of silastic sheets overlay on the graft. While myringoplasty outcomes are widely reported, few studies examine institutional learning and behavioral adaptation over time. This current study investigates the impact of the 2019 guidelines changes on surgical practice and whether this translates into anatomical and audiometric improvement. This offers the possibility to verify the appropriateness of critical quality investigations in our daily practice.

## Methods

### Study goal

The primary objective of this study was to assess whether the alterations of practice guidelines derived from the 2019 study translated into measurable changes in clinical practice and patient outcomes [4]. To evaluate this, the primary outcome of this study was to assess the adherence to the updated surgical recommendations. Accordingly, the number of surgeries annually performed per surgeon, the graft used for reparation of the tympanic membrane, and the use of silastic sheets were analyzed and compared to the 2019 results.

The secondary outcome of this study was defined as the anatomical success rate of the myringoplasty. Tympanic closure was classified as successful if a closed tympanic membrane was noted throughout the whole follow-up period. Tympanic closure was classified as unsuccessful if there was either a re-perforation, pinpoint perforation, or if a reoperation was needed during the follow-up period.

The tertiary outcome was the auditory performance, measured by the amount of improvement of the air–bone gap (ABG) after surgery.

### Study design and study population

In order to perform a comparative quality evaluation and assess change in practice, the same methodology as our previous study in 2019 was applied [4].

This retrospective follow-up study included patients who underwent myringoplasty surgery during the period from January 2020 up until December 2021 in Jeroen Bosch Hospital (JBZ) in the Netherlands.

Patient selection was performed using a treatment-code for myringoplasty. Patients who underwent a tympanoplasty other than type I, mastoidectomy, ossicular reconstruction, or cholesteatoma surgery were excluded. Patients with a follow-up of less than two months were excluded as well. All the remaining patients who underwent myringoplasty surgery during the study period were included, regardless of whether the procedure was primary or secondary.

### Ethical considerations

This retrospective follow-up analysis examines outcomes in a cohort of patients. Patients were diagnosed and treated according to departmental and national guidelines. This research is of negligible risk and involves the use of existing patient data. Furthermore, there is no harm and/or dis- comforts for the participants.

All the data were collected as part of an internal evaluation to improve quality of care. The main risk in this retrospective cohort was the breach of trust or confidentiality. We minimized this risk by making the data only accessible for the researchers and data was anonymized as soon as feasible. We believed that the expected benefits of this project would exceed this minimal amount of inconvenience.

### Data collection and classification

Data were collected on gender and age, the side of the perforation, the type of perforation, the size of perforation, the number of ear infections in the year before the surgery, and previous ear surgery. The size of the perforation could either be small (1 quadrant), subtotal (2 or 3 quadrants) or complete. Concerning the surgery itself, data about the surgeon, the surgical approach, applied graft material, the presence of myringosclerosis, and the intraoperative use of silastic sheets were collected. Surgeons 1, 2, and 3 were involved in the decision-making process regarding the myringoplasty policy that preceded the start of this study. Surgeons 4, 5, and 6 were visiting senior residents that operated with a high degree of independence, but final responsibility maintained with the supervising surgeon. Data about the follow-up length (in months), the use of postoperative antibiotics, and complications were collected. Complications were defined as peroperative or postoperative adverse events that could negatively impact surgical outcomes.

Surgical approaches were classified according to the type of incision described in each surgical report. Endaural incision was categorized as endaural approach and retro-auricular incision was classified as retro-auricular approach. Endomeatal incisions or graft placement through the perforation without the use of any of these incisions were classified as transcanal approach. Additionally, the placement of cartilage grafts over a denuded umbo, using the over-under myringoplasty technique, was documented. With this technique, a cartilage graft is placed medial to the remaining tympanic membrane and lateral to the malleus handle, which allows direct contact with the denuded umbo [5].

Grafts were placed by underlay technique and supported by gel foam (Willospon) and covered by gel foam. In some cases, silastic sheets were used as onlay. When a biflanged cartilage plug was used, it was placed in the perforation and covered with gel foam. Antibacterial drops and postoperative antibiotics were prescribed on indication. General anesthesia was used in all operations. A distinction was made between primary and secondary (revision) surgery.

Additionally, both pre- and postoperative audiometry were assessed. Audiograms were acquired using Affinity, Interacoustics. The mean bone and air conduction thresholds were calculated (500 Hz, 1000 Hz, 2000 Hz and 4000 Hz). The mean ABG was calculated from these thresholds.

### Statistical analysis

Data analysis was performed using IBM SPSS Statistics (v30.0.0.0, IBM Corp.; Armonk, NY, USA). Chi-squared test and Fisher’s exact test were performed to assess the differences in success rates of the determinants. An exploratory binary logistic regression was conducted to examine whether key variables (graft type, surgeon group, complications) were associated with higher probability of successful closure of the tympanic membrane.

Both an independent T-test and paired T-test were used to evaluate the auditory outcome. A one-way ANOVA was performed to compare mean ABG gain across different variables.

This study considered *p*-values lower than 0.05 (*p* < 0.05) as statistically significant.

## Results

After exclusion, 172 myringoplasties were included in this analysis. The surgical characteristics affecting success rate of myringoplasties and the comparison with the 2019 results are presented in Table 1. Characteristics of the patient population are shown in Table 2. The overall follow-up time was 9.8 ± 9.3 months, ranging from 2 months to 37 months. Patients with a successful tympanic membrane closure had a mean follow-up time of 9.1 ± 9.3 months. The mean follow-up time for patients with an unsuccessful tympanic closure was 14.5 ± 8.0 months.

### Adaptation of recommendations

Surgeons 1 to 3 were involved in the cohort study of 2019. According to these recommendations, they performed at least 15 procedures per year, (52, 67 and 23 resp.). The other surgeons mentioned were residents visiting the clinic for a shorter period. The required number of 15 procedures per year was therefore not always reached. In 2019, 80 cases received surgery with the use of only fascia for myringoplasty. In 2024, this was reduced to 4 cases as there was an overall shift to the graft types of cartilage, cartilage+, and butterfly. The use of silastic sheets was reduced from 80 cases in 2019 to 13 cases in 2024 (Table 1).

### Success rate of tympanic closure

The overall success rate of closure of the perforation was 88.4% compared to 74.9% in 2019. In total 148 primary interventions were performed, of which 90.5% were successful tympanic closures. 24 revision surgeries were included with an overall success rate of closure of 75.0% (Table 2). 20 patients had an unsuccessful tympanic closure (Table 1).

Success rates were highest in surgeon 1 with a success of 92.3% (Table 1). The three most experienced surgeons collectively had a success rate of 89.4%, compared to 83.3% in the group of other surgeons that consisted of visiting residents. Yet, changes in success rate were noted and no significant difference in the success rate of tympanic closure was found between the experienced surgeon group vs. other surgeons.

**Table 1:** Comparison of results 2019 and 2024

Factor	2019 (4)					2024				
	Number of patients (%)	Successful tympanic closure (% successful)	Primary surgery or revision	Material used for closure	Use of silastic sheets	Number of patients (%)	Successful tympanic closure (% successful)	Primary surgery or revision	Material used for closure	Use of silastic sheets
Total	195 (100)	**146 (74.9)**		Missing: 2 Cartilage: 41 Butterfly: 55 **Fascia: 80** Cartilage+: 8 Others: 9		172 (100)	**152 (88.4)**		Missing: 0 Cartilage: 68 Butterfly: 73 **Fascia: 4** Cartilage+: 27	
Surgeon*										
1	54 (27.7)	50 (92.6)	-	Cartilage: 35 Butterfly: 18 Fascia: 0 Cartilage+: 0	Yes: 21 No: 33	52 (30.2)	48 (92.3)	Primary: 44 Revision: 8	Cartilage: 43 Butterfly: 9 Fascia: 0 Cartilage+: 0	None
2	53 (27.2)	34 (64.2)	-	Cartilage: 2 Butterfly: 13 Fascia: 25 Cartilage+: 6	Yes: 48 No: 5	67 (39.0)	68 (86.6)	Primary: 60 Revision: 7	Cartilage: 4 Butterfly: 37 Fascia: 1 Cartilage+: 25	Yes: 13 No: 54
3	36 (18.4)	28 (77.8)	-	Cartilage: 0 Butterfly: 7 Fascia: 25 Cartilage+: 2	None	23 (13.4)	21 (91.3)	Primary: 19 Revision:4	Cartilage: 10 Butterfly: 12 Fascia: 1 Cartilage+: 0	None
Others	52 (26.7)	34 (65.4)	-	Cartilage: 4 Butterfly: 17 Fascia: 30 Cartilage+: 0	Yes: 11 No: 41	32 (17.4)	25 (83.3)	Primary: 25 Revision: 5	Cartilage: 11 Butterfly: 15 Fascia: 2 Cartilage+: 2	None

*Order of surgeons used in 2024

**Table 2:** Patient characteristics affecting success rate of myringoplasties

Factor	Number of patients (%)	Successful tympanic closure (%)	*P* value	Significant
Gender				
Female	80 (46.5)	72 (90.0)	0.535	NS
Male	92 (53.5)	80 (87.0)		
Age				
Children (0–17 year)	85 (49.4)	73 (85.9)	0.314	NS
Adults (18 and older)	87 (50.6)	79 (90.8)		
Number of ear infections				
0	126 (73.3)	115 (91.3)	0.099	NS
1–2	44 (25.6)	35 (79.5)		
>2	2 (1.2)	2 (100.0)		
Myringoplasty				
Primary	148 (86.0)	134 (90.5)	**0.028**	**S**
Revision	24 (14.0)	18 (75.0)		
Previous ear surgery				
Yes	112 (65.1)	99 (88.4)	0.991	NS
No	60 (34.9)	53 (88.3)		
Surgical technique				
Transcanal	161 (93.6)	142 (88.2)	0.567	NS
Endaural	6 (3.5)	6 (100)		
Retro-auriculair	5 (2.9)	4 (80.0)		
Graft above umbo				
Yes	23 (13.4)	**22 (95.7)**	**0.020**	**S**
No	71 (41.3)	57 (80.3)		
Does not apply	78 (45.3)	73 (93.6)		
Postoperative eardrops				
Yes	23 (13.4)	19 (82.6)	0.354	NS
No	149 (86.6)	133 (89.3)		
Complications				
Yes	8 (4.7)	**3 (37.5)**	**< 0.001**	**S**
No	164 (95.3)	149 (90.9)		

Overall successful tympanic closure increased from 74.9% in 2019 to 88.4% in 2024. The use of fascia graft decreased after 2019, showing an increase in cartilage, cartilage+, and butterfly graft. *Fascia corresponds with the use of temporalis fascia; cartilage + corresponds with the use of cartilage together with the use of temporalis fascia* [4].

### Characteristics affecting closure success rate

A chi-squared test was performed to assess the differences in closure success rate between patient characteristics. There was a significant correlation between the occurrence of complications and the overall success rate of tympanic closure (Table 2). Additionally, primary surgery was significantly associated with higher successful closure rates compared to revision surgery (90.5% vs. 75.0%). Furthermore, a significant improvement in closing results was found with the placement of a cartilage graft over a denuded umbo using the over-under technique. This variable was newly introduced in the present analysis and was not included in the 2019 dataset [4], thus being interpreted as a secondary, post hoc finding.

No significant differences were found for the following variables: gender, age, number of ear infections in the year prior to surgery, previous ear surgery, the side, size or site of perforation, the surgeon, the attendance of a resident, surgical technique, graft used, the use of silastic sheets or the use of medical eardrops (Table 2).

An exploratory binary logistic regression was conducted to assess whether graft type, surgeon group, and presence of complications were associated with higher probability of successful tympanic membrane closure. Only the variable ‘complications’ significantly predicted this outcome, with patients experiencing complications having lower odds of successful closure (OR = 0.053, *p* < 0.001). These reported complications included postoperative otorrhea (*n* = 2), a non-integrated ventilation tube (*n* = 2), anterior displacement of cartilage (*n* = 1), anterior displacement of cartilage with reperforation (*n* = 1), cartilage extrusion (*n* = 1), and postoperative infection (*n* = 1). Graft type and surgeon group were not significant predictors of successful closure (*p* = 0.194 and *p* = 0.443). The results of this analysis should be interpreted with caution due to the low number of failures that limit statistical power.

Significant associations were found for graft above umbo, primary vs. revision surgery, and the presence of complications. Patients with a graft above the umbo using the over-under technique had the highest success rate (95.7%). Primary surgery was associated with higher successful closure rates than revision surgery (90.5% vs. 75.0%). Complications were associated with a decrease in success rate (37.5%).

### Pre- and post-surgery audiometry

Both a paired T-test and an independent T-test were performed to analyze differences between pre- and post-surgery audiometry. A significant difference was found between the pre-surgery and post-surgery audiometry results in air-conduction (8.54 dB ± 8.45. *p* < 0.001), as well as for the air-bone gap (8.19 ± 9.83. *p* < 0.001). Additionally, postoperative air conduction gain was significantly higher in cases of successful anatomical closure than in cases of unsuccessful closure (9.06 ± 8.48 dB; 4.14 ± 6.96 dB. *p* = 0.027). There was no significant audiometric gain for bone conduction or ABG (Table 3).

Additionally, a one-way ANOVA showed a significant difference in mean ABG improvement between surgeons (F = 2.72, p = 0.022). Post hoc analysis with Tukey’s test revealed a significant difference in ABG gain between surgeons 2, one of the surgeons with most experience, and surgeon 4, a visiting resident (mean difference = 7.30 dB, p = 0.048). This indicates that patients operated on by surgeon 2 had more improvement in audiometry than patients operated on by surgeon 4. Pairwise comparison showed no other significant results between surgeons.

**Table 3:** Mean audiometry results from pre- and post-surgery and after successful and unsuccessful tympanic closure

	Audiometry results (dB ± SD)		Audiometric gain^*^ (dB ± SD)	*P* value
	Pre-operative	Post-operative		
Air conduction (*n* = 152)	26.09 ± 11.01	17.54 ± 12.33	**8.54 ± 8.45**	**< 0.001**
Bone conduction (*n* = 147)	5.24 ± 9.37	4.78 ± 10.31	0.46 ± 7.43	0.457
Air-bone gap (*n* = 152)	21.02 ± 8.50	12.83 ± 9.24	**8.19 ± 9.83**	**< 0.001**
	Audiometric gain^*^ (dB ± SD)			*P* value
	Successful closure	Unsuccessful closure		
Air conduction	**9.06 ± 8.48**	**4.14 ± 6.96**		**0.027**
Bone conduction	0.59 ± 7.57	− 0.69 ± 6.13		0.528
Air-bone gap	8.54 ± 9.87	5.26 ± 9.26		0.209

* A positive audiometric gain indicates improved hearing.

No other significant difference in ABG gain was found within the variables: location of the perforation, size of the perforation, surgical approach, or graft type. 

Significant audiometric improvement was observed for air conduction and air-bone gap postoperatively. Patients with successful tympanic closure showed significantly greater audiometric gain in air conduction than those with unsuccessful closure.

### Handling of missing data

The presence of myringosclerosis was not consistently documented, which resulted in this variable being excluded from the analysis.

Due to incomplete surgical reporting, data on the location of the perforation were missing in 16 cases. Regarding the hearing results, 21 cases had missing air conduction data, and 24 cases had missing bone conduction data. This could mainly be attributed to the absence of a post-surgery audiometry test due to the restrictions concerning the Covid-19 pandemic or patients not showing up for their appointment. In 1 out of the 21 cases, the audiometry report was missing. Because of the Covid-19 pandemic, the timeframe in which the pre- and post-surgery hearing tests had to be performed was extended.

To examine whether the missing perforation location and audiometry data could have introduced systematic bias, a sensitivity analysis was performed. All cases with missing perforation location data (*n* = 16) were compared to those with complete perforation data (*n* = 156). Respectively, all cases with missing audiometry data either before or after the surgery (*n* = 24) were compared to cases with complete audiometry data (*n* = 148). T-test or Chi-squared test was performed for different variables: age, sex, surgeon, graft material, and successful closure. The only statistically significant association was found between the surgeon and the presence of missing data on the location of the perforation (χ²(5) = 27.17, *p* < 0.001), confirming that surgical reporting varied between surgeons. No other significant associations were found for the location of the perforation. No significant differences were found for the audiometry data either, suggesting that the missing data occurred randomly across the population.

## Discussion

The primary objective of this study was to assess whether the changes in practice guidelines implemented after the 2019 study translated into measurable changes in clinical practice and patient outcomes. The primary outcome to evaluate this was to assess the adherence to the updated surgical recommendations. Comparison with the 2019 study shows an overall shift in surgical behavior following the implementation of updated guidelines. While recommended in 2019, the required number of 15 procedures per year was not always reached due to residents performing operations during their training period. Additionally, use of temporalis fascia was reduced from 80 cases in 2019 to 4 in 2024. The use of silastic sheets was reduced from 80 cases in 2019 to 13 cases in 2024 [4].

The secondary outcome was to assess the anatomical success rate of the myringoplasty following these changes in guidelines. The overall success rate of closure of the perforation increased to 88.4% compared to 74.9% in 2019.

The tertiary outcome was to assess auditory performance, measured by the amount of improvement of the air–bone gap (ABG) after surgery. Air conduction and the air–bone gap improved significantly after surgery, with a mean ABG improvement of 8.19 ± 9.83 dB.

### Impact of guidelines changes

After the revision of clinical guidelines in 2019, notable alterations in surgical approach and material selection can be observed. There was an overall shift to the use of cartilage and butterfly material instead of temporal fascia. This transition showed higher closure success rates in myringoplasty, which may be due to temporalis fascia being thinner, more flexible and less resilient [6].

Furthermore, the number of operations performed by surgeons with a smaller number of myringoplasties per year decreased from 27.0% in 2019 to 17.4% in 2024. Less experienced operators were often residents who performed fewer procedures during a brief period. Consequently, they had fewer opportunities to develop technical proficiency in myringoplasty compared to more experienced surgeons. While acknowledging the importance of surgical training for skill development of visiting residents, the reduced involvement of these lower-volume operators in routine surgeries may reflect a shift towards optimizing surgical outcomes for patients.

The three most experienced myringoplasty surgeons collectively had an overall success rate of 89.4% compared to 83.3% of the other surgeons. While the difference between these two groups is not statistically significant, variation at the individual surgeon level could still have clinical relevance. Our results suggest that evaluating the qualities of each surgeon, such as their individual surgical technique, patient selection, and experience with different graft materials can provide valuable insights into improving overall myringoplasty outcomes. Additionally, they show that higher surgical exposure and experience contribute to improved procedural outcomes.

### Guideline changes and functional hearing outcomes

The adaptation of these changes in guidelines significantly improved the success rate of the surgery, thereby reducing the need for revision surgery. However, the functional outcomes for patients did not show a similar improvement, particularly for the differences in hearing acuity. While the air conduction thresholds improved significantly after surgery, the air–bone gap did not show a significant difference between patients with successful versus unsuccessful closure. This finding is consistent with previous research, which shows that in patients with successful tympanic closure, the degree of ABG improvement varied widely and a substantial proportion of patients showed minimal functional gain [7]. One possible explanation is that the size of the perforation may have decreased in cases classified as unsuccessful. While re-perforation occurred, the residual perforation could have been smaller than the original, allowing for partial improvement in sound conduction. As a result, audiometric outcomes, such as ABG, could still show improvement.

Furthermore, comparison to the 2019 results shows that successful tympanic closure does not directly translate into improved functional outcomes. Despite the increased anatomical closure rate after the changed guidelines implementations (88.4% in 2024 vs. 74.9% in 2019), there was no improvement in terms of ABG gain among successfully closed cases (8.54 dB in 2024 vs. 10.10 dB in 2019). The overall shift from the use of temporalis fascia to the application of cartilage could contribute to this as cartilage is generally stiffer and heavier, thus potentially decreasing sound transmission [6, 8]. However, recent studies found no statistical difference in ABG gain between fascia and cartilage grafts [5, 8, 9]. Yet, both the comparison with the 2019 results, as the similar ABG gain in successful vs. unsuccessful closures, suggest that surgery success rates alone may not solely account for the improvement of hearing.

### Effectiveness of implementation

While research considering collaborative quality improvement of care has been conducted in the past [10], this study was the first to implement the principles of internal self-evaluation in this field. The involvement of physicians is essential to ensure these changes in behavior after critical internal evaluation.

This study showed that ENT-surgeons in this hospital were open to quality control and self-assessment, causing greater effectiveness and a higher likelihood of producing sustained improvements in clinical practice. However, for the adoption of external developed guidelines, 45% of the otorhinolaryngologists were non-adherent to the recommendations [11]. The effectiveness of implementing different surgical strategies depends on several factors, including positive intercollegiate interactions, strong senior leadership, and effective communication, as reported in previous studies [12]. Moreover, surgeons, practice groups, and organizations were more likely to change if they had sufficient knowledge of a procedure, if data showed patient outcomes would be improved or if they conducted research on a different procedure themselves [12, 13]. Therefore, creating an environment that encourages continuous learning and effectively researches proposed guideline changes is essential for successful adaptation to revised surgical recommendations.

### Strengths and limitations

A strength of this study was the replicability with the 2019 results. Using the same methodology and protocol allowed comparison with previous results, showing trends of changing surgical behavior [4]. Furthermore, this study used a large sample size of 172 patients, which is similar to the 195 of the 2019 study and allowed for analysis across a diverse patient population, thereby increasing the reliability of our results.

However, it is important to acknowledge the limitations of this study. Firstly, these findings are based on a retrospective cohort study. While we controlled for known variables, confounding variables and selection bias could not be excluded. Future prospective studies could help confirm these associations and allow for stronger causal conclusions. Secondly, the follow-up durations differed between the successful and unsuccessful tympanic closure groups (9.1 ± 9.3 months and 14.5 ± 8.0 months). Due to the retrospective nature of this study, a standardized follow-up period was not possible. Therefore, the shorter follow-up in the successful closure group may have introduced survival bias. Thirdly, this study was restricted to a single hospital. Findings based on a larger number of participating hospitals would enhance the generalizability of the data, supporting nation-wide conclusions rather than limiting interpretation to changes within a single hospital. However, the significance of these results and large sample size shows promising perspective for larger future application. Finally, the follow-up regarding audiometry data was not complete due to missed appointments and the Covid-19 pandemic. While this is a limitation, we believe the overall impact on our conclusions was minimal.

### Implications for clinical practice

This study demonstrated that identifying areas of improvement through critical internal quality evaluation facilitated consistent adherence to newly implemented guidelines. Hence, we advocate continuous implementation of critical internal quality evaluation in daily care. Hospitals and healthcare professionals should support this implementation by adjusting electronic patient records to facilitate systematic data collection and develop dashboards to monitor and analyze surgical practices and outcomes.

Facilitation of critical internal quality evaluation could subsequently improve accountability. By engaging in self-assessment, clinicians hold themselves accountable for the quality of care they provide. This personal accountability fosters a stronger commitment to providing high-quality health care.

## Conclusion

This study evaluated the clinical impact of implementing the revised 2019 surgical guidelines for myringoplasty procedures. Internal self-assessment of surgical outcomes was associated with changes in surgical behavior, material preferences and subsequently an increase in success rates of tympanic closure. These data suggest that a critical internal quality investigation could significantly alter surgeon practices. Improvements can rapidly be incorporated into routine practice and subsequently re-evaluated in daily practice. Therefore, internal critical examination is a valuable tool for clinicians to enhance the quality of their clinical practice, continually improve patient care, and contribute to the overall quality of healthcare services.

## Data Availability

The data that support the findings and conclusions of this study are available and can be requested from M.H. van Lier. The data are not publicly available due to privacy considerations.

## Code availability

This article does not contain any material or code requiring sharing.
